# Dr. John P. Colegrove

**Published:** 1916-02

**Authors:** 


**Dr. John P. Colegrove,** Buffalo 1875, died at his home in Salamanca Jan. 14, aged 83. During his later years he had been an oil producer.

